# On the analysis of clonogenic survival data: Statistical alternatives to the linear-quadratic model

**DOI:** 10.1186/s13014-016-0584-z

**Published:** 2016-01-28

**Authors:** Steffen Unkel, Claus Belka, Kirsten Lauber

**Affiliations:** Department of Medical Statistics University Medical Centre, Georg-August-University Goettingen, Goettingen, Germany; Clinic for Radiotherapy and Radiation Oncology, LMU Munich, Munich, Germany; Clinic Cooperation Group ‘Personalized Radiotherapy in Head and Neck Cancer’, Helmholtz Center Munich, Munich, Germany

**Keywords:** Clonogenic survival, Colony formation assays, Hierarchical clustering, Linear-quadratic model, Non-linear regression, Principal component analysis, Radiotherapy

## Abstract

**Background:**

The most frequently used method to quantitatively describe the response to ionizing irradiation in terms of clonogenic survival is the linear-quadratic (LQ) model. In the LQ model, the logarithm of the surviving fraction is regressed linearly on the radiation dose by means of a second-degree polynomial. The ratio of the estimated parameters for the linear and quadratic term, respectively, represents the dose at which both terms have the same weight in the abrogation of clonogenic survival. This ratio is known as the *α*/*β* ratio. However, there are plausible scenarios in which the *α*/*β* ratio fails to sufficiently reflect differences between dose-response curves, for example when curves with similar *α*/*β* ratio but different overall steepness are being compared. In such situations, the interpretation of the LQ model is severely limited.

**Methods:**

Colony formation assays were performed in order to measure the clonogenic survival of nine human pancreatic cancer cell lines and immortalized human pancreatic ductal epithelial cells upon irradiation at 0-10 Gy. The resulting dataset was subjected to LQ regression and non-linear log-logistic regression. Dimensionality reduction of the data was performed by cluster analysis and principal component analysis.

**Results:**

Both the LQ model and the non-linear log-logistic regression model resulted in accurate approximations of the observed dose-response relationships in the dataset of clonogenic survival. However, in contrast to the LQ model the non-linear regression model allowed the discrimination of curves with different overall steepness but similar *α*/*β* ratio and revealed an improved goodness-of-fit. Additionally, the estimated parameters in the non-linear model exhibit a more direct interpretation than the *α*/*β* ratio. Dimensionality reduction of clonogenic survival data by means of cluster analysis was shown to be a useful tool for classifying radioresistant and sensitive cell lines. More quantitatively, principal component analysis allowed the extraction of scores of radioresistance, which displayed significant correlations with the estimated parameters of the regression models.

**Conclusions:**

Undoubtedly, LQ regression is a robust method for the analysis of clonogenic survival data. Nevertheless, alternative approaches including non-linear regression and multivariate techniques such as cluster analysis and principal component analysis represent versatile tools for the extraction of parameters and/or scores of the cellular response towards ionizing irradiation with a more intuitive biological interpretation. The latter are highly informative for correlation analyses with other types of data, including functional genomics data that are increasingly beinggenerated.

## Background

Clonogenic survival is an important endpoint to measure the cellular response towards ionizing irradiation *in vitro*. It is commonly assessed by 2D or 3D colony formation assays, which are based on the capacity of single cells to grow to colonies consisting of at least 50 cells [1–3]. Accordingly, cells retaining the capacity to undergo at least 5–6 rounds of cell division in response to irradiation are quantified. These clonogenic cells usually constitute a rather small subpopulation that is further reduced upon irradiation. All other cells within the population are considered as reproductively dead or inactive. Colony formation assays are frequently utilized to examine and characterize differences in sensitivity towards ionizing irradiation between tumor and normal cells and to assess the impact of additional treatments and/or manipulations on the radiation response. Whereas the abrogation of clonogenic survival is of utmost importance for tumor control and for the prevention of recurrences, preservation of continued proliferation and clonogenic survival are a crucial prerequisite for maintaining integrity and function in normal tissue.

**Fig. 1:**
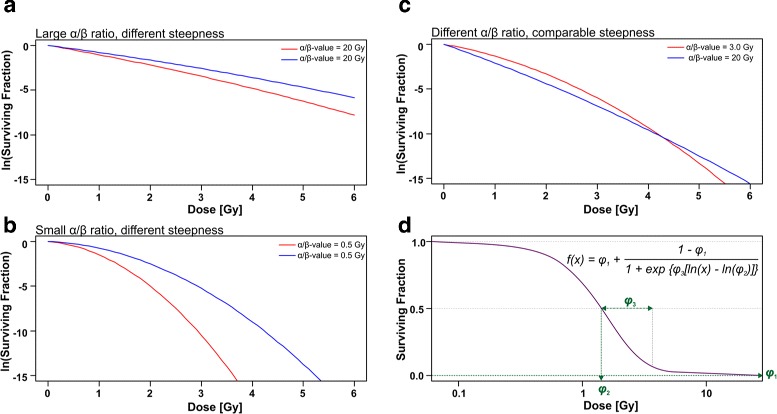
The linear-quadratic model can differentiate clonogenic survival curves with varying contributions of the linear and the quadratic terms but fails to reflect the overall steepness of the curves. Three-parameter log-logistic regression represents an interesting alternative. Hypothetical clonogenic survival curves with different *α*/*β* ratio and overall steepness were constructed. Curves with identical *α*/*β* ratio but different steepness are shown for *α*/*β*=20 Gy (**a**) and *α*/*β*=0.5 Gy (**b**). In (**c**), curves with different *α*/*β* ratio (3 or 20 Gy, respectively), but similar overall steepness are depicted. **d** Three-parameter log-logistic model with parameters *ϕ*_1_, *ϕ*_2_ and *ϕ*_3_

For the measurement of clonogenic survival, cells are seeded in appropriate dilutions, subjected to ionizing irradiation at different doses, and 1–3 weeks later colonies are fixed, stained, and counted. To calculate the surviving fraction at a given dose, the number of colonies is divided by the number of seeded cells and normalized to the plating efficiency of the not irradiated controls. The log-transformed values of the surviving fractions are plotted against the corresponding irradiation doses, and traditionally linear-quadratic (LQ) regression analysis is performed, which describes the function of the surviving fraction by a second-degree polynomial with a linear and a quadratic term (see Eq. (1)) [4]. The coefficients and their *α*/*β* ratio reflect the weights of the linear and quadratic term upon the reduction in clonogenic survival. When *α*≫*β*, the function of the surviving fraction reveals a basically linear character (Fig. 1a). In contrast, when *β*≫*α*, the curvature dominates the function (Fig. 1b). Hence, the *α*/*β* ratio is well suited to differentiate curves with more linear or more quadratic shape, respectively (Fig. 1c). Nevertheless, it fails to assess the overall steepness of the curve, which is obviously related to radiosensitivity and/or resistance (Fig. 1a and b). Additionally, the *α*/*β* ratio lacks an intuitive biological interpretation. For many applications, it would be very helpful to have parametric information and/or quantitative scores, which reflect the overall survival curve with greater explanatory depth.

In principle, clonogenic dose response datasets reveal sigmoidal character, thus rendering them suitable for non-linear regression analysis [5]. In this study, a multi-parameter equation with a log-logistic transformation of the predictor, i.e. the irradiation dose, was employed (Fig. 1d and Eq. (3)). The parameters *ϕ*_*i*_ exhibit a high degree of biological transparency, including the asymptotic clonogenic survival at infinite irradiation dose, the irradiation dose at the inflection point of the curve, and a measure related to its steepness (Fig. 1d). To the best of our knowledge, a comprehensive comparison of LQ and non-linear log-logistic regression analysis has not been performed with clonogenic survival data so far. This is the aim of the present study.

Moreover, categorizing cells as sensitive or resistant with respect to ionizing irradiation might be helpful for multiple applications when studying the cellular responses towards ionizing irradiation as well as their underlying mechanisms. Quantitative scores of radioresistance and/or sensitivity with the power to reflect clonogenic survival over the whole dose range analyzed would bear strong informative value for correlation analyses with datasets of gene expression profiling and other “deep information datasets” that are increasingly being collected. Two methods of dimensionality reduction, which allow these calculations, are hierarchical cluster analysis and principal component analysis (PCA) [6, 7].

These considerations inspired us to perform the present study. Clonogenic survival data of eight human pancreatic cancer cell lines and immortalized human pancreatic ductal epithelial cells (HPDE) upon ionizing irradiation were subjected to regression analyses with the LQ and the non-linear log-logistic model as well as dimensionality reduction by hierarchical clustering and PCA. The results were compared, and the strengths and weaknesses of the respective algorithms are discussed. Finally, we propose a combination of all these methods as a workflow for the identification and validation of targets for biologically motivated improvements of cancer radiotherapy [8].

## Methods

### Cell lines

The human pancreatic cancer cell lines Capan-2, Dan-G, and FamPac were obtained from Cell Lines Service (Heidelberg, Germany). PaTu-8988T, Panc-1, MiaPaca-2, L3.6pl (a metastastic subclone of Colo 357), Suit-2 007 (a metastastic subclone of Suit-2) and immortalized human pancreatic ductal epithelial cells (HPDE) were kindly provided by Maximilian Schnurr, Department of Clinical Pharmacology, LMU Munich [9, 10]. Identity of cell lines was confirmed by short tandem repeat (STR) typing (service provided by the DSMZ, Braunschweig, Germany). Tumor cell lines were cultured in DMEM (Capan-2, PaTu-8988T, Panc-1, MiaPaca-2, L3.6pl, and Suit-2 007) or RPMI-1640 medium (FamPac and Dan-G) supplemented with 10 % heat-inactivated fetal calf serum (FCS), 100 U/ml penicillin, and 0.1 mg/ml streptomycin (all from Life Technologies, Karlsruhe, Germany) at 37 °C and 7.5 % CO_2_, or 5 % CO_2_, respectively. HPDE cells were maintained in a 1:1 mixture of keratinocyte serum-free medium and RPMI-1640 medium supplemented with 250 ng/ml human epidermal growth factor (EGF), 25 *μ*g/ml bovine pituitary gland extract (BPE), 5 % FCS, 50 U/ml penicillin, and 0.05 mg/ml streptomycin (all from Life Technologies) at 37 °C and 5 % CO_2_.

### Colony formation assays

Clonogenic survival was examined in colony formation assays. Cells were seeded as single cell suspensions into 6-well plates in a range of 100–100,000 cells per well in order to yield approximately 50 colonies per well depending on the different irradiation doses applied. Upon adherence for 4 h, cells were irradiated, and colony formation was allowed for 14 days. Subsequently, fixation and staining was performed in 80 % ethanol containing 0.3 % (w/v) methylene blue (both from Sigma-Aldrich, Taufkirchen, Germany), and colonies with more than 50 cells were counted. The number of colonies was divided by the number of seeded cells and normalized on the plating efficiency of the not irradiated controls. Data from 3–4 independent experiments were used for the statistical analyses.

### Computations

Computations for this study were carried out using the R Software, version 3.2.1 [11]. All computer code used is available upon request.

### Linear regression framework

Recall the LQ model

(1) $$\begin{array}{@{}rcl@{}} y_{ij} & = & \exp\left(\alpha x_{ij} + \beta x_{ij}^{2} \right)\exp\left(\epsilon_{ij}\right)  \\ \Leftrightarrow \ln\left(y_{ij}\right) & = & \alpha x_{ij} + \beta x^{2}_{ij} + \epsilon_{ij} \enspace, \end{array} $$

where *x*_*ij*_ denotes the radiation dose for cell line *i*$(i=1,\dots,n)$ and replicate *j*$(j=1,\dots,J_{i})$, *y*_*ij*_ are the resulting survival fractions (SFs) of cells and *ε*_*ij*_ are error terms with $\epsilon _{\textit {ij}} \sim \mathcal {N}\left (0,\sigma ^{2}\right)$. Clonogenic survival of all *n* cell lines was measured over the same range of radiation doses *x*=0,1,2,4,6,8 and 10 Gy. The semi-log regression model (1) is linear in the unknown parameters *α* and *β* that are to be estimated from the data. The goodness-of-fit of the model (1) can be evaluated by means of the coefficient of determination, denoted *R*^2^.

### Non-linear regression framework

In addition to the LQ model, we fitted regression models of the form

(2) $$ y_{ij} = f(x_{ij}; \phi_{1},\dots,\phi_{p}) + \epsilon_{ij} \enspace,  $$

where the mean function *f* is non-linear in one or more of the *p* parameters $\phi _{1},\dots,\phi _{p}$. In particular, consider the following log-logistic mean function for sigmoidal curves (see e.g. [12]):

(3) $$ f(x) = \phi_{1} +\frac{1-\phi_{1}}{\left(1+\exp\{\phi_{3}[\ln(x)-\ln(\phi_{2})] \}\right)^{\phi_{4}}} \enspace,  $$

where *ϕ*_1_ is the horizontal asymptote as *x*→*∞*, *ϕ*_2_ is the inflection point of the curve at which the response is midway between 1 and *ϕ*_1_, and the parameter *ϕ*_3_ is proportional to the slope d *f*(*u*)/d*u* at *u*_0_ in the log-logistic mean function *f*(*u*) with *u*= ln(*x*) and *u*_0_= ln(*ϕ*_2_). The function is not symmetric (on the log scale) for *ϕ*_4_ different from 1, hence *ϕ*_4_ may be called an asymmetry parameter. We have chosen the log-logistic mean function (3) to ensure that *f*(*x*)=1 for *x*=0 Gy. Instead, the logistic mean function $f(x) = \phi _{1} +\frac {1-\phi _{1}}{\left (1+\exp \{\phi _{3}[x-\phi _{2}] \}\right)^{\phi _{4}}}$ would be different from 1 at a dose of 0 Gy. We performed model selection comparing four nested models with mean function (3): the least restrictive four-parameter model with parameters $\phi _{1},\dots,\phi _{4}$, a three-parameter model with *ϕ*_4_ fixed at one, a two-parameter model with *ϕ*_4_ fixed at one and lower asymptote *ϕ*_1_ fixed at zero and a one-parameter model with *ϕ*_4_=1, *ϕ*_1_=0 and *ϕ*_3_=1. For model selection we used the Akaike information criterion (AIC), which is defined as follows [13]:

(4) $$ {\text{AIC}} = 2 (p+1) - 2 \ln(\mathcal{L}) \enspace,  $$

where $\mathcal {L}$ denotes the maximized value of the likelihood function for the model. The AIC rewards goodness of fit as assessed by the likelihood function, but also includes a penalty that is an increasing function of the number of estimated parameters. Given a set of candidate models for the data, the preferred model is the one with the minimum AIC value. However, the final model choice was based on both statistical and biological grounds. The chosen non-linear model was compared to a more general analysis of variance (ANOVA) model. The ANOVA model imposes no restrictions on how the response changes from one dose level to another, as there will be one parameter for each dose level. That is, the non-linear model is a submodel of the ANOVA model and we used an *F*-test [5] to test the null hypothesis that the ANOVA model can be simplified to the non-linear regression model. Note that the most common interpretation of *R*^2^ for linear regression does not hold true for non-linear regression. In the non-linear case, the *R*^2^ value is not the amount of variability in the dependent variable explained by the independent variable. In other words, in a non-linear regime the total sum-of-squares is not equal to the regression sum-of-squares plus the residual sum-of-squares (RSS). Therefore, *R*^2^ should not be used as a goodness-of-fit measure in non-linear regression [14]. Instead one can use the residual variance $\hat {\sigma }^{2} = \frac {{\text {RSS}}}{df}$, where the circumflex denotes the estimated value of *σ*^2^ and *df* denotes the degrees of freedom for the model. Alternatively, one can use the residual standard error $\hat {\sigma }=\sqrt {\hat {\sigma }^{2}}$ as a summary measure for the model fit [15]. We also devoted ourselves to model diagnostics checking whether substantive departures from the model assumptions can be found.

### Cluster analysis

Cluster analysis of the clonogenic survival data was employed in order to classify radioresistant and sensitive cell lines [6]. Given the 9×6 data matrix **X**, which consists of mean survival fractions (in %) of the 9 cell lines measured at 6 radiation doses 1, 2, 4, 6, 8 and 10 Gy, a 9×9 distance matrix **D** was calculated using the Euclidean distance as a proximity measure. The matrix **D** was then analyzed by means of agglomerative hierarchical clustering, which produces a series of partitions of the data: the first consists of 9 single-member “clusters”; the last consists of a single group containing all 9 cell lines. At each stage, cell lines were fused that are closest according to Ward’s method [16] in which the fusion of two clusters is based on the size of an error sum-of-squares criterion. We investigated the sensitivity of the results with respect to the clustering method. The obtained classifications are represented by a dendrogram, which illustrates the fusions made at each stage of the analysis. To provide further guidance for determining the number of clusters we present a silhouette plot, which is a means of assessing the quality of a cluster solution enabling one to identify “poorly” classified objects and so distinguishing clear-cut clusters from weak ones. More details on the interpretation of the silhouette plot are given in the “Results” section.

### Principal component analysis

Principal component analysis (PCA) allows the extraction of scores of radioresistance for the cell lines under investigation [7]. Suppose without changing the notation that the columns of **X** have been mean-centered and scaled to unit variance. Then, the 6×6 sample correlation matrix is computed as **C**=**X**^⊤^**X**/(*n*−1) with **X**^⊤^ being the transposed matrix of **X**. The eigendecomposition of **C** can be written as

(5) $$ {\textbf{C}} = {\textbf{V}} {\boldsymbol{\Lambda}} {\textbf{V}^{\top}}~,  $$

where ***Λ*** is a diagonal matrix with the eigenvalues of **C** sorted in decreasing order, *λ*_1_≥*λ*_2_≥…≥*λ*_6_≥0, on its main diagonal and **V** is an orthogonal matrix whose columns ${\textbf {v}_{1}},\dots,{\textbf {v}_{6}}$ are the unit-norm eigenvectors of $\lambda _{1},\dots,\lambda _{6}$. The matrix **V** is composed of coefficients or loadings and and the *r*-th sample principal component (PC) with mean zero and variance *λ*_*r*_ is $\textbf {z}_{r} = {\textbf {X}} \textbf {v}_{r}(r=1,\dots,6)$. Rescaled loadings are calculated as $\textbf {v}_{r}^{*} = \sqrt {\lambda _{r}} \textbf {v}_{r}$ for which $\textbf {v}_{r}^{*}{{\!~\!}^{\top }} \textbf {v}_{r}^{*} = \lambda _{r}$, rather than unity. For standardized data, this rescaling leads to coefficients that are the correlations between the components and the original variables. Various criteria for choosing the optimal number *R* of uncorrelated principal components (PCs) to be retained do exist [7]. For example, one proposal is to retain the first *R* components which explain a large proportion, (*λ*_1_+…+*λ*_*R*_)/(*λ*_1_+…+*λ*_6_), of the total variation in the data, say 70–80 %. Another is to retain only components (for standardized data) that possess eigenvalues greater than one, which is known as Kaiser’s rule [17].

## Results

Clonogenic survival of eight human pancreatic cancer cell lines and immortalized human pancreatic ductal epithelial cells (HPDE) was measured by colony formation assays upon exposure to ionizing irradiation, and the resulting dataset was subjected to different computational analyses in order to obtain measures of radioresistance and/or sensitivity.

### LQ model

**Fig. 2:**
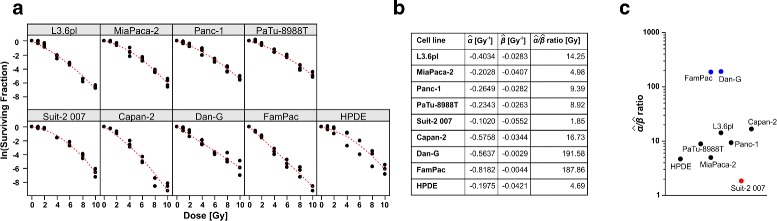
Linear-quadratic regression analysis of clonogenic survival data obtained from eight human pancreatic cancer cell lines and immortalized human pancreatic ductal epithelial cells. Cells were subjected to colony formation assays upon irradiation at 0-10 Gy. After irradiation, cells were incubated for 14 days, the numbers of colonies with more than 50 cells were counted, and the surviving fractions were calculated. Results of 3–4 independent experiments are depicted in a Trellis plot of the log-transformed survival fraction versus radiation dose with fitted regression curves superimposed (**a**). Estimated coefficients along with the $\hat {\alpha }/\hat {\beta }$ ratios for the LQ model (**b**, **c**)

First, the most common method, the LQ model, was employed to fit the clonogenic survival data. Here, the log-transformed surviving fraction (SF) at a given irradiation dose is approximated by a second-degree polynomial with a linear and a quadratic term. A Trellis plot of the log-transformed survival fractions versus the irradiation doses was constructed, and the fitted regression curves were superimposed (Fig. 2a). For all cell lines, a very good agreement between the observed data and the estimated regression curves was obtained indicating that the LQ model is capturing the systematic part in the data very well. The goodness-of-fit of the LQ model, as evaluated by means of *R*^2^, varied between 95.46 % for HPDE and 99.32 % for FamPac cells (average 98.52 %). The estimated coefficients along with their $\hat {\alpha }/\hat {\beta }$ ratios showed that the weights of the linear and quadratic term differ substantially across the cell lines (Fig. 2b). Whereas the linear term did not reach statistical significance at the 5 % level for Suit-2 007 cells, this applied to the quadratic term in case of Dan-G and FamPac cells. Accordingly, in these three cell lines, the residual term dominated the reduction in clonogenic survival upon irradiation (the quadratic term in case of Suit-2 007, and the linear term in case of Dan-G and FamPac, respectively). The $\hat {\alpha }/\hat {\beta }$ ratios ranged from 1.85 Gy (Suit-2 007) to 191.58 Gy (Dan-G), and their graphical display disclosed three groups of cell lines (Fig. 2c). Given that the $\hat {\alpha }/\hat {\beta }$ ratio is traditionally considered as a measure of radiosensitivity [4], this classified Suit-2 007 cells as particularly radioresistant and FamPac as well as Dan-G cells as highly radiosensitive. The majority of pancreatic cancer cell lines exhibited $\hat {\alpha }/\hat {\beta }$ ratios between 4 and 16 Gy, which were comparable to or even higher than that of the non-malignant HPDE cells ($\hat {\alpha }/\hat {\beta }= 4.69$ Gy). Hence, our clonogenic survival data do not confirm the common opinion that pancreatic cancer cells exhibit an extraordinarily high degree of radioresistance as compared to normal cells – at least *in vitro* [18, 19]. As anticipated, LQ regression of the clonogenic survival data and the resulting $\hat {\alpha }/\hat {\beta }$ ratios revealed the major limitation in the sense that assessing radioresistance via the $\hat {\alpha }/\hat {\beta }$ ratio cannot differentiate survival curves of similar $\hat {\alpha }/\hat {\beta }$ ratio but discrepant steepness. This was exemplified in case of Capan-2 and L3.6pl cells. Whereas the $\hat {\alpha }/\hat {\beta }$ ratio was rather similar ($\hat {\alpha }/\hat {\beta } = 16.73$ Gy for Capan-2, and $\hat {\alpha }/\hat {\beta } = 14.25$ Gy for L3.6pl, respectively), the overall steepness of the curves was obviously different characterizing Capan-2 cells as clearly more radiosensitive than L3.6pl cells. In the following, different approaches were used in order to overcome this shortcoming and to extract parameters and/or scores of the radiation response, which are superior to the $\hat {\alpha }/\hat {\beta }$ ratio in terms of reflecting radiosensitivity and which have a more direct and intuitive biological interpretation.

### Non-linear regression

**Fig. 3:**
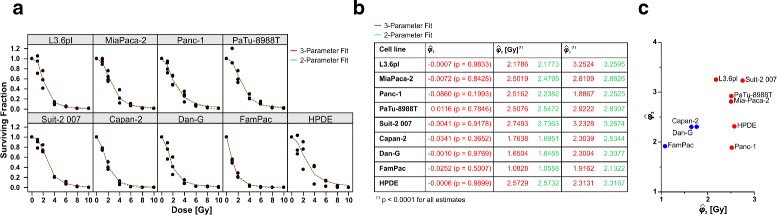
Non-linear log-logistic regression of clonogenic survival data provides parameters with intuitive biological interpretation. A three- and a two-parameter log-logistic regression model were fitted to the clonogenic survival data. Trellis plot of the survival fraction versus radiation dose with fitted regression curves superimposed (**a**, red for the three-parameter, green for the two-parameter model). Estimated regression parameters and *p*-values for the log-logistic regression model (**b**); $\hat {\phi }_{1}$ represents the survival fraction for which the dose approaches infinity, $\hat {\phi }_{3}$ is a slope factor that refers to the steepness of the curve, and $\hat {\phi }_{2}$ represents the irradiation dose that indicates a surviving fraction of 0.5 (ED_50_). **c** Plot of the cell lines in the space of $\hat {\phi }_{2}$ and $\hat {\phi }_{3}$

Next, the clonogenic survival data were analyzed by means of non-linear log-logistic regression, which utilizes a multi-parameter equation for sigmoidal curves (Fig. 1d). Table 1 displays the log-likelihood, the number of estimated parameters (*p*+1), and the Akaike information criterion (AIC) for each of the four nested non-linear log-logistic regression models. Naturally, the least restrictive model with four parameters gave the best fit to the data (in terms of the likelihood). However, for the clonogenic survival dataset, this model contained too many parameters, and particularly the asymmetry parameter *ϕ*_4_ appeared redundant. Indeed, in terms of the AIC, models that fix *ϕ*_4_ at 1 were superior (Table 1). On the other hand, reducing the model to the standard one-parameter log-logistic model proved not appropriate for the given dataset. The remaining alternatives of a two- or three-parameter model gave virtually equal results. The AIC for the two-parameter model with the lower asymptote *ϕ*_1_ (reflecting the surviving fraction at infinite irradiation dose) fixed at 0 was slightly lower than that of the three-parameter model. However, we consider the latter to be the most suited one for our dataset, because in general the lower asymptote should be allowed to be either positive or zero for very high irradiation doses. The estimated regression curves obtained by fitting the chosen three-parameter model superimposed to the measured clonogenic survival data are shown in the Trellis plot (Fig. 3a). For comparison, the fitted regression curves of the two-parameter model are also depicted. Visually, there was a good fit between the observed data and the estimated regression curves. The overall residual standard errors of the fitted three-parameter and two-parameter models were $\hat {\sigma } = 0.0756$ and $\hat {\sigma } = 0.0746$, respectively, confirming that the sigmoidal curves are capturing the variation in the data very well. Notably, the residual standard error of the LQ model was $\hat {\sigma } = 0.4336$. The estimated parameters $\hat {\phi }_{1}$, $\hat {\phi }_{2}$ and $\hat {\phi }_{3}$ along with their *p*-values are shown in Fig. 3b; $\hat {\phi }_{1}$ was never significantly different from zero indicating that clonogenic survival upon irradiation at infinite does converges to 0. For all cell lines, the scale factor $\hat {\phi }_{3}$ was greater than 1 implying that the curves were steeper than the standard log-logistic curve. The irradiation dose, which resulted in 50 % survival and which is commonly referred to as median effective dose ED_50_ [12], is represented by $\hat {\phi }_{2}$. It varied between 1.08 Gy (FamPac) and 2.75 Gy (Suit-2 007) with a mean of 2.17 Gy. Plotting the results of the different cell lines in the pace of $\hat {\phi }_{2}$ and $\hat {\phi }_{3}$ suggested this time that the cell lines split into 2 groups. Hence, the distribution was somewhat different from the distribution according to the $\hat {\alpha }/\hat {\beta }$ ratios as obtained by the LQ model. FamPac, Dan-G, and Capan-2 cells formed a group of radiosensitive cell lines with low $\hat {\phi }_{2}$ values (ED_50_), and low to intermediate $\hat {\phi }_{3}$ values that are related to the steepness of the curve. On the other hand, high $\hat {\phi }_{2}$ values and intermediate to high $\hat {\phi }_{3}$ values were estimated for the radioresistant group comprising L3.6pl, Suit-2 007, HPDE, PaTu-8988T, MiaPaca-2, and Panc-1 cells. With *ϕ*_2_ (ED_50_), the three-parameter log-logistic model provides a parameter that is more intuitive and has a more direct biological interpretation than the rather abstract *α*/*β* ratio of the LQ model. Additionally, log-logistic regression enabled the discrimination of more sensitive Capan-2 and more resistant L3.6pl cells. Thus, the three-parameter log-logistic model appears to be superior to the LQ model in terms of deriving parameters of radiosensitivity with more direct biological interpretation and more quantitative depth – at least in the given dataset.

**Table 1:** Log-likelihood, number of estimated parameters and AIC for four nested non-linear logistic regression models fitted to the Pancreas data

Model	$\ln (\mathcal {L})$	*p*+1	AIC
Mean function (3)	258.24	37	−442.48
*ϕ*_4_=1	249.59	28	−443.18
*ϕ*_4_=1, *ϕ*_1_=0,	247.06	19	−456.11
*ϕ*_4_=1, *ϕ*_1_=0, *ϕ*_3_=1	80.17	10	−140.34

In order to compare the performance of the three-parameter log-logistic model to a more general ANOVA model, an *F*-test was performed. The lack-of-fit test was overwhelmingly non-significant (*p*-value 0.9824) further strengthening the suitability of the non-linear regression model. We also investigated other sigmoidal relationships between the surviving fraction and the irradiation dose to assess the mean structure in non-linear models. For example, the maximized log-likelihood of the three-parameter Weibull model was 237.77 compared to 249.59 of our chosen model (Table 1) indicating that this model did not fit the data better than the log-logistic model presented here. In conclusion, our results identify the three-parameter log-logistic model as highly appropriate for the analysis of clonogenic survival data.

### Hierarchical clustering

By plotting the $\hat {\alpha }/\hat {\beta }$ ratios of the fitted LQ model or the values for $\hat {\phi }_{2}$ and $\hat {\phi }_{3}$ of the fitted three-parameter log-logistic model, we already attempted to define groups of cell lines with different radiosensitivity (Figs. 2c and 3c). However, the grouping decisions were made merely by visual inspection, and thus were rather subjective. A more objective approach for classification is hierarchical clustering, which was applied to our dataset in the following.

**Fig. 4:**
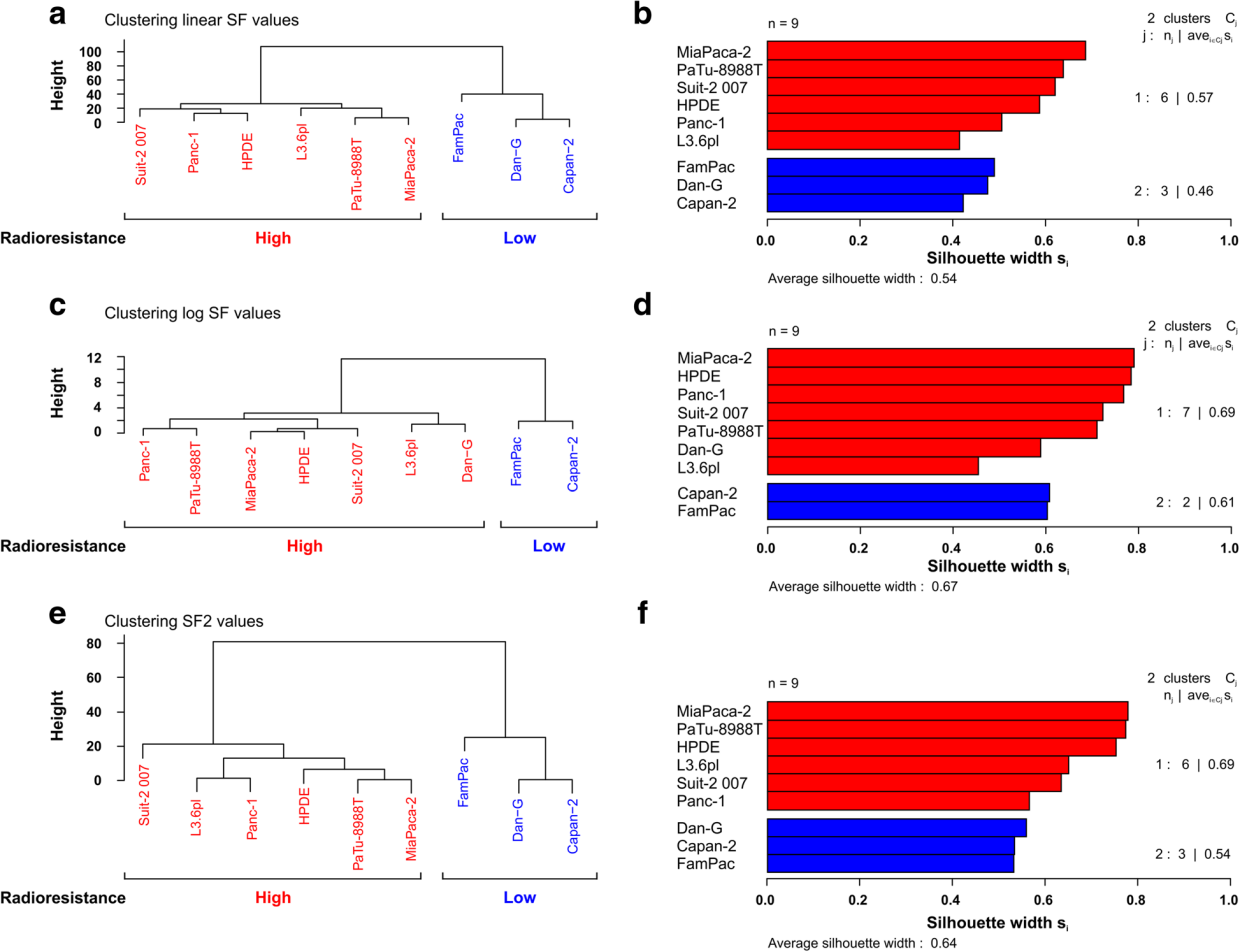
Cluster analysis of the clonogenic survival data discloses groups of radioresistant and sensitive cells. Euclidean distances of the nine cell lines were analyzed by means of agglomerative hierarchical clustering of original SF data (**a**, **b**), log-transformed SF data (**b**, **c**), and SF2 values only (**d**, **e**). Dendrograms (**a**, **c**, **e**): a two-cluster solution with a group of radioresistant and a group of sensitive cell lines can be identified. The silhouette plots (**b**, **d**, **f**) display a good quality of the obtained two-cluster solution

At first, hierarchical clustering was performed with the original clonogenic survival fractions. The obtained dendrogram illustrating the process of the agglomerative hierarchical clustering, and the partitions produced at each stage are displayed in Fig. 4a. The nodes in this diagram represent clusters, and the heights represent the distances at which each fusion is made. Large changes in fusion levels are considered to indicate the best cut of the tree, and thus suggest the number of clusters. The dendrogram of the clonogenic survival data revealed a clear structure that displayed two main groups with different radiosensitivity. Whereas cluster 1 comprised the more sensitive cell lines FamPac, Dan-G, and Capan-2, all other cell lines were located in cluster 2. A two-cluster solution was also observed when employing the non-hierarchical partitioning around medoids (PAM) cluster algorithm [20] (results omitted). As a means of evaluating the clustering process, silhouette plots are commonly employed [6]. The silhouette plot of the two-cluster solution obtained by the best cut of the hierarchical algorithm and PAM displays for each object (cell line) an index *s*_*i*_∈ [ −1,1], called a silhouette (Fig. 4b). When *s*_*i*_ has a value close to 1, object *i* is taken “well classified”. When *s*_*i*_ is close to -1, object *i* is taken to be “misclassified”. When the index is near zero it is not clear whether the object should have been assigned to its current cluster or a neighboring one. In Fig. 4b the *s*_*i*_ are displayed as horizontal bars, ranked in decreasing order for each cluster. The average silhouette width of all nine cell lines was 0.54, which can be considered a reasonable classification [20].

Notably, the obtained clusters were highly similar to the groups derived from the three-parameter log-logistic model (Fig. 3c). Moreover, in contrast to the $\hat {\alpha }/\hat {\beta }$ ratio of the LQ model, cluster analysis in fact was able to differentiate more sensitive Capan-2 from more resistant L3.6pl cells. We also performed cluster analysis on the log-transformed clonogenic survival data. The corresponding results were similar to the ones obtained by clustering the original survival fractions with the difference that Dan-G cells were allocated to the cluster with the radioresistant cell lines (Fig. 4c,d). Clonogenic survival at 2 Gy (SF2) is widely used as a measure of radiosensitivity. Therefore, we also performed cluster analysis with the SF2 values only. The results were virtually identical to the ones obtained by categorizing the full range of SF values (Fig. 4e,f). Interestingly, the two clusters were more clearly separated as compared to the clusters generated from the full range of SF data. This result is due to the fact that the SF2 clustering takes into account the survival at a single dose only and cannot reflect the observation that some cell lines appear sensitive in the higher dose range and resistant in the lower dose range and vice versa. Overall, we consider hierarchical clustering of clonogenic survival data as a versatile tool to categorize radiosensitive and radioresistant cell lines for further analyses. This might be of relevance for the selection of radioresistant and sensitive cell lines for studies aiming to delineate molecular mechanisms of the cellular response towards ionizing irradiation, and particularly for identifying and characterizing signaling pathways of resistance and/or hypersensitivity.

### Principal component analysis

Hierarchical clustering of clonogenic survival data can serve as a method for categorizing radioresistant and sensitive cell lines. Yet, it does not provide a quantitative measure of radioresistance and/or sensitivity. Principal component analysis (PCA) is a method that offers this possibility. It utilizes orthogonal transformations in order to convert a set of correlated variables into a derived set of linearly uncorrelated variables, called principal components (PCs).

The sample correlation matrix of the clonogenic survival data was

(6) $$ {\textbf{C}} = \left(\begin{array}{cccccc} 1.00 & 0.83 & 0.66 & 0.76 & 0.58 & 0.58 \\ 0.83 & 1.00 & 0.82 & 0.84 & 0.66 & 0.33 \\ 0.66 & 0.82 & 1.00 & 0.91 & 0.86 & 0.54 \\ 0.76 & 0.84 & 0.91 & 1.00 & 0.88 & 0.68 \\ 0.58 & 0.66 & 0.86 & 0.88 & 1.00 & 0.81 \\ 0.58 & 0.33 & 0.54 & 0.68 & 0.81 & 1.00 \\ \end{array} \right).  $$

**Fig. 5:**
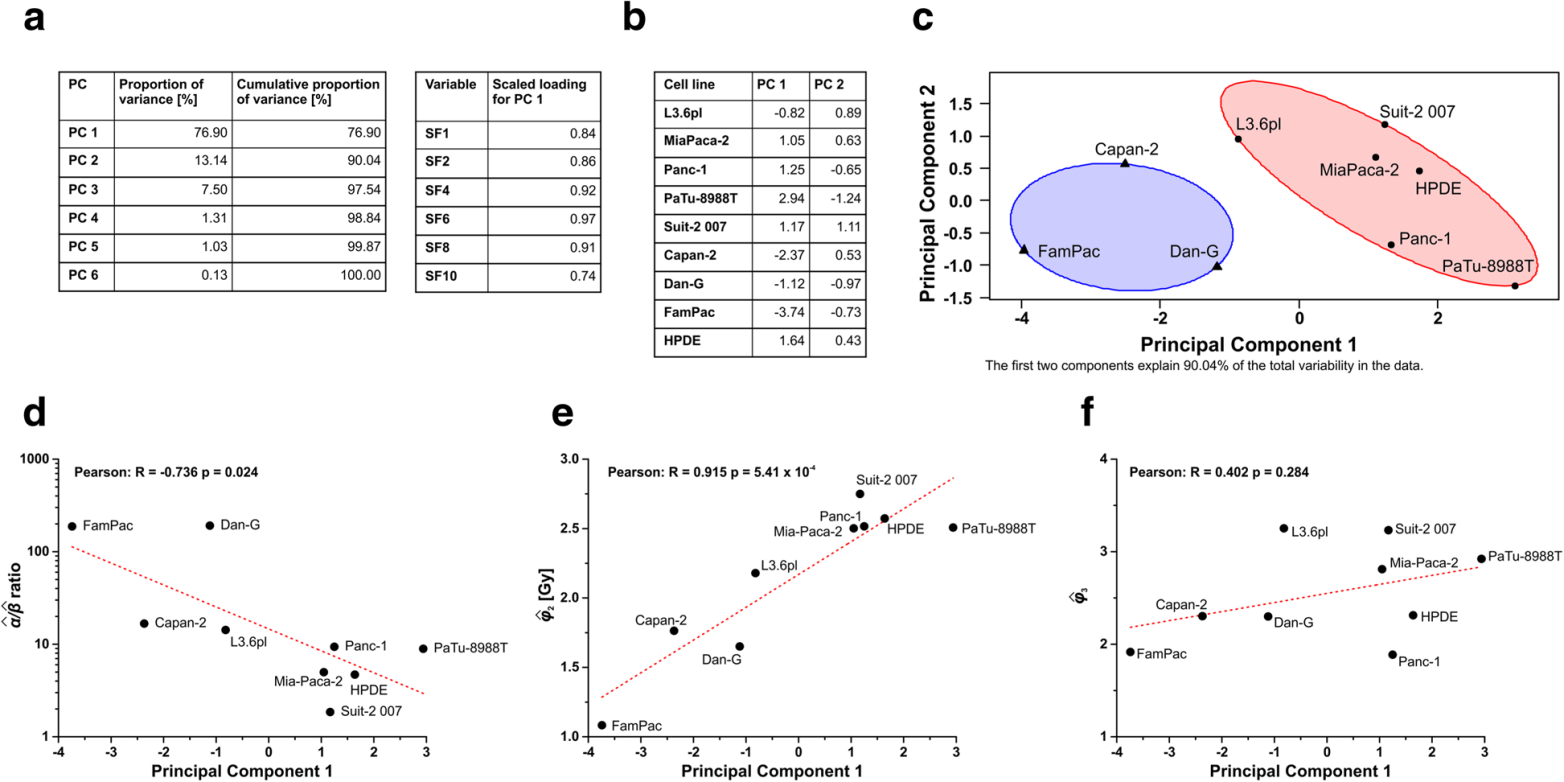
Principal component analysis of the clonogenic survival data enables the extraction of scores of radioresistance. Standardized clonogenic survival data were subjected to principal component analysis (PCA). In (**a**), the proportions of variance for the six PCs and the scaled loadings of the variables on the first PC that accounts for 76.9 % of the total variability in the data are shown. The first PC exhibits well-balanced loadings for the measured variables. The scores of the nine cell lines on the first two PCs are presented in (**b**). The resulting two-cluster solution is displayed in the space of the first two PCs that reflect 90.04 % of the total variability in the data (**c**). Pearson correlation analyses of the first PC were conducted with the estimated parameters extracted from the fitted regression models (**d**, **e**, **f**)

The correlation matrix (6) demonstrated moderately to strongly positive, linear correlations between all survival fractions. Hence, PCA appeared to be an adequate tool for dimensionality reduction of these data. The extracted first PC alone accounted for 76.9 % of the total variability in the data and was the only PC whose eigenvalue was greater than one (Fig. 5a). Thus, according to Kaiser’s rule [17], it was sufficient to retain only the first PC. The rescaled loadings $\textbf {v}_{1}^{*}$ indicated a high degree of correlation with the extracted PC for all six surviving fractions (SF1-SF10) implying that the first PC represents a well-balanced measure of clonogenic survival over the whole dose range that was analyzed (Fig. 5a). The corresponding scores of the first PC for all cell lines are shown in Fig. 5b. They ranged between 2.94 in case of radioresistant PaTu-8988T cells, and −3.74 for radiosensitive FamPac cells. Indeed, the scores of the first PC can be interpreted as a weighted index of radioresistance within the dose range measured. A plot of the data projected into the subspace of the first two PCs (for better visualization) with the two-cluster classification solution superimposed is depicted in Fig. 5c. It clearly confirms the two-cluster solution of radiosensitive and resistant cell lines that was obtained by hierarchical cluster analysis and shows the scores of the first PC as a quantitative measure of radioresistance. In order to compare the PCA results to the results obtained by LQ and log-logistic regression, correlation analyses between the extracted parameters and the first PC were performed. A significant negative correlation was observed between the $\hat {\alpha }/\hat {\beta }$ value and the first PC supporting the common use of the *α*/*β* ratio as a(n) (inverse) measure of radioresistance. Intriguingly, an even better, yet positive correlation was obtained between $\hat {\phi }_{2}$ and the first PC, again strengthening its versatility as an indicator of radioresistance. In terms of completeness, the correlation between $\hat {\phi }_{3}$ and the first PC was calculated resulting in the absence of statistical significance (Fig. 5d–f).

## Discussion

Polynomial models, such as the LQ model, are linear in the parameters. Fitting polynomial models can lead to an accurate approximation to the true regression function, their application for the analysis of clonogenic survival data being no exception. However, these models are empirical in the sense that they are only based on the observed relationship between the response and the predictors and do not include mechanistic considerations *a priori* [21]. For the LQ model, molecular theories of the underlying biological mechanisms were originally provided in the 1970s and have been refined later on [22–25]. Based on the assumption that clonogenic cell death upon ionizing irradiation derives from lethal DNA damage, which is predominantly represented by DNA double strand breaks (DSBs), the linear term in this model was attributed to DSBs resulting from single irradiation events, whereas the quadratic term was interpreted as DSBs resulting from two independent irradiation events. Yet, the parameters derived from LQ regression (*α*, *β*, and the *α*/*β* ratio) lack biological transparency, and in the present study they were shown to be inferior to the parameters derived from log-logistic regression in terms of explanatory power. We are well aware that from the LQ model one can easily compute ED_50_ and other values with direct biological interpretability. However, in the radiobiological routine this is simply not done. Instead, *α*/*β* ratios are commonly used as measures of the cellular response towards ionizing irradiation. And these clearly lack biological interpretability.

Unlike linear polynomial models, non-linear regression models are often based on a theory for the mechanism accounting for the response. In consequence, the model parameters in a non-linear model have a more plastic interpretation [21]. In case of the three-parameter log-logistic regression model used here, the curve of the surviving fraction takes on a sigmoidal shape between 1 and the lower asymptote *ϕ*_1_ that corresponds to the surviving fraction for positively infinite dose; *ϕ*_3_ is related to the steepness of the curve, and *ϕ*_2_ corresponds to the irradiation dose at which the survival fraction reaches 0.5 (ED_50_). Other effective doses, such as ED_10_ or ED_90_, may be easily obtained from the fitted regression curve as well. Moreover, constraints can be built into a non-linear model easily and are harder to enforce for linear models. If, for instance, the response attains an asymptotic value as the dose grows, the non-linear models have such a built-in behavior automatically. We decided in favor of log-logistic regression instead of logistic regression to ensure that the response is equal to 1 for 0 Gy. A three-parameter model was selected in order to preserve the possibility of SF>0 for infinite irradiation doses, which may be observed for cellular subpopulations with absolute radioresistance.

The iterative nature of the fitting algorithm, which requires a set of user-supplied starting values, could be considered as a disadvantage of non-linear models. However, many software programs have built-in self-starter functions, which substitute for manually searching the starting values [15]. Non-linear regression models have successfully been used for describing dose-response dependencies for a long time [26]. They perform with a high degree of accuracy and provide intuitive biological interpretations. The present study shows that they also have their merits in describing the cellular response towards ionizing irradiation in colony formation assays. For the given dataset, the goodness-of-fit, as evaluated by means of the residual standard error, was even superior to the LQ model. It should be noted that fitting a model to the original survival data (as is the case for non-linear regression) emphasizes the low dose range with high survival fractions. In contrast, fitting a model to log-transformed survival data (as is the case for the LQ model) focuses on the higher dose range with low survival fractions. Given that the applicability of the LQ model in the higher dose range is being controversially discussed [27], and the clinically relevant dose range is 1–4 Gy, we do not consider this bias towards the lower dose range as a shortcoming but rather as an advantage of the non-linear regression model.

We have also employed dimensionality reduction techniques, namely cluster analysis to classify radioresistant and sensitive cell lines, and PCA that allows the extraction of scores of radioresistance. Note that these two techniques should not be considered as substitutes of regression models. Regression models and dimensionality reduction techniques have different purposes and provide complementary information. Whereas linear and non-linear regression models aim at quantitatively describing the relationship between a response and one or more predictors using a statistical model, dimensionality reduction techniques aim at summarizing the observed data in a lower-dimensional space. Nevertheless, often there is a benefit in analyzing the data from more than one angle, as a single technique is seldom able to reveal all important features in a given set of data. In this regard, our study suggests that a combination of hierarchical clustering, PCA, and regression of clonogenic survival data represents a promising approach for target identification in the context of biologically motivated improvements of cancer radiotherapy. Very recently, we have successfully used the aforementioned methods and identified HSP90 as a candidate molecule for targeted radiosensitization of resistant soft tissue sarcoma [28]. Cluster analysis was employed to classify radioresistant and sensitive cell lines from a panel of human soft tissue sarcoma cell lines. PCA-derived scores of radioresistance were applied to correlation analyses with transcriptomic data of the DNA damage response resulting in the identification of HSP90 and its client proteins ATM, ATR, and NBS1 as candidate mediators of radioresistance. Their functional involvement was addressed by pharmacological HSP90 inhibition, and the impact on clonogenic survival was finally examined and quantified by regression analyses.

## Conclusions

The present study was performed in order to exemplify the versatility of non-linear regression and dimensionality reduction via hierarchical clustering and PCA as statistical alternatives to the LQ model for the analysis of clonogenic survival data. A combination of these techniques represents a powerful toolkit for the characterization of clonogenic cell death upon ionizing irradiation as well as the extraction of parameters and/or scores with quantitative explanatory power and direct biological interpretation. Together with results from transcriptomic, proteomic and other “big data” endeavors, these scores might be utilized to identify candidate targets and/or pathways for molecular manipulation of cellular responses towards ionizing irradiation as well as their functional validation.

## References

[1] Franken NA, Rodermond HM, Stap J, Haveman J, van Bree C (2006). Clonogenic assay of cells in vitro. Nat Protoc.

[2] Puck TT, Marcus PI (1956). Action of x-rays on mammalian cells. J Exp Med.

[3] Soffar A, Storch K, Aleem E, Cordes N (2003). Cdk2 knockdown enhances head and neck cancer cell radiosensitivity. Int J Radiat Biol.

[4] Franken NA, Oei AL, Kok HP, Rodermond HM, Sminia P, Crezee J (2013). Cell survival and radiosensitisation: modulation of the linear and quadratic parameters of the lq model (review). Int J Oncol.

[5] Bates DM, Watts DG (2007). Nonlinear Regression Analysis and Its Applications.

[6] Everitt BS, Landau S, Leese M, Stahl D (2012). Cluster Analysis.

[7] Jolliffe IT (2002). Principal Component Analysis.

[8] Orth M, Lauber K, Niyazi M, Friedl AA, Li M, Maihöfer C (2014). Current concepts in clinical radiation oncology. Radiat Environ Biophys..

[9] Bruns CJ, Harbison MT, Kuniyasu H, Eue I, Fidler IJ (1999). In vivo selection and characterization of metastatic variants from human pancreatic adenocarcinoma by using orthotopic implantation in nude mice. Neoplasia.

[10] Iwamura T, Caffrey TC, Kitamura N, Yamanari H, Setoguchi T, Hollingsworth MA (1997). P-selectin expression in a metastatic pancreatic tumor cell line (SUIT-2). Cancer Res.

[11] R Core Team (2015). R: A Language and Environment for Statistical Computing.

[12] Ritz C, Streibig JC (2005). Bioassay analysis using R. J Stat Soft.

[13] Akaike H (1974). A new look at the statistical model identification. IEEE Trans Autom Control.

[14] Spiess AN, Neumeyer N (2010). An evaluation of *R*^2^ as an inadequate measure for nonlinear models in pharmacological and biochemical research: a monte carlo approach. BMC Pharmacol.

[15] Ritz C, Streibig JC (2008). Nonlinear Regression with R.

[16] Ward JH (1963). Hierarchical groupings to optimze an objective function. J Am Stat Assoc.

[17] Kaiser HF (1960). The application of electronic computers to factor analysis. Educ Psychol Meas.

[18] Schlaich F, Brons S, Haberer T, Debus J, Combs SE, Weber KJ (2013). Comparison of the effects of photon versus carbon ion irradiation when combined with chemotherapy in vitro. Radiat Oncol.

[19] Schnurr M, Duewell P, Bauer C, Rothenfusser S, Lauber K, Endres S (2015). Strategies to relieve immunosuppression in pancreatic cancer. Immunother.

[20] Kaufman L, Rousseuw PJ (2005). Finding Groups in Data: An Introduction to Cluster Analysis.

[21] Pinheiro JC, Bates DG (2000). Mixed-Effects Models in S and S-Plus.

[22] Franken NA, Barendsen GW (2014). Enhancement of radiation effectiveness by hyperthermia and incorporation of halogenated pyrimidines at low radiation doses as compared with high doses: implications for mechanisms. Int J Radiat Biol.

[23] Franken NA, ten Cate R, Krawczyk PM, Stap J, Haveman J, Aten J (2011). Comparison of rbe values of high-let *α*-particles for the induction of dna-dsbs, chromosome aberrations and cell reproductive death. Radiat Oncol.

[24] Chadwick KH, Leenhouts HP (1973). A molecular theory of cell survival. Phys Med Biol.

[25] Kellerer AM, Rossi HH (1972). The theory of dual radiation action. Curr Top Radiat Res Q.

[26] Govindarajulu Z (2001). Statistical Techniques in Bioassay.

[27] Ekstrand KE (2010). The Hug-Kellerer equation as the universal cell survival curve. Phys Med Biol.

[28] Ernst A, Anders H, Kapfhammer H, Orth M, Hennel R, Seidl K (2015). Hsp90 inhibition as a means of radiosensitizing resistant, aggressive soft tissue sarcomas. Cancer Lett.

